# Mendelian Randomization Analysis Reveals No Causal Genetic Link Between Dentofacial Anomalies and Dental Caries

**DOI:** 10.3290/j.ohpd.c_2319

**Published:** 2025-11-04

**Authors:** Xiaomeng Wang, Qi Fan, Beidi Ma, Jinhan Yu, Gongjie Yuan

**Affiliations:** a Xiaomeng Wang Dentist, Department of Stomatology, Shanghai Children’s Hospital, School of Medicine, Shanghai Jiao Tong University, Shanghai, China. Performed the studies, participated in collecting data, drafted the manuscript, performed the statistical analysis and participated in its design, acquisition, analysis, and interpretation of data, drafted the manuscript, read and approved the final manuscript.; b Qi Fan Dentist, Department of Stomatology, Shanghai Children’s Hospital, School of Medicine, Shanghai Jiao Tong University, Shanghai, China. Performed the studies, participated in collecting data, drafted the manuscript, read and approved the final manuscript.; c Beidi Ma Dentist, Department of Stomatology, Shanghai Children’s Hospital, School of Medicine, Shanghai Jiao Tong University, Shanghai, China. Performed the statistical analysis and participated in its design, read and approved the final manuscript.; d Jinhan Yu Dentist, Department of Stomatology, Shanghai Children’s Hospital, School of Medicine, Shanghai Jiao Tong University, Shanghai, China. Performed the statistical analysis and participated in its design, read and approved the final manuscript.; e Gongjie Yuan Professor, Department of Stomatology, Shanghai Children’s Hospital, School of Medicine, Shanghai Jiao Tong University, Shanghai, China. Acquisition, analysis, or interpretation of data, drafted the manuscript, read and approved the final manuscript.

**Keywords:** causal effect, dental caries, deposits on teeth, malocclusion, Mendelian randomization.

## Abstract

**Purpose:**

Dentofacial anomalies are closely linked to dental health, including caries and periodontal disease. This study examined the potential causal relationship between genetic variations associated with dental anomalies, such as malocclusion, and the risk of dental caries.

**Materials and Methods:**

A two-sample Mendelian randomization (MR) using genome-wide association studies (GWAS) data was conducted. Dental caries data were obtained from the UKB and GWAS catalog, while dental anomaly data came from FinnGen R12. The primary analysis used inverse-variance weighted (IVW) methods, with weighted median, MR-Egger, and weighted models for validation. Horizontal pleiotropy and outliers were assessed via MR-Egger and MR-PRESSO, while Cochran’s Q test evaluated heterogeneity. Leave-One-Out (LOO) analysis identified predominant instrumental variables (IVs).

**Results:**

The genetic prediction results indicated no statistically significant causal associations between dentofacial anomalies and dental caries (for all three cohorts, p>0.05). Also, IVW indicated no causal associations between dentofacial anomalies and other health problems, including mouth ulcers, toothache, loose teeth, bleeding gums, acute and chronic periodontitis, and painful gums. However, for the outcome of loose teeth, analysis revealed evidence of heterogeneity and suggested potential horizontal pleiotropy, with rs79490532 identified as a outlier. After removing rs79490532, the estimated causal effect of dentofacial anomalies on loose teeth remained statistically non-significant.

**Conclusion:**

Our findings suggest that dentofacial anomalies, including malocclusion, do not have a direct genetic impact on dental health. These results emphasize the importance of prioritizing oral hygiene practices, dietary interventions, and targeted preventive strategies over corrective orthodontic approaches in clinical management to improve dental health outcomes.

Oral health is a critical yet often overlooked component of global health. Dental problems, such as tooth decay, gum disease, and tooth loss, affect billions worldwide, severely impacting overall well-being, productivity, and healthcare systems.^26^ Caries, also known as tooth decay or cavities, is the most common dental health issue affecting teeth and a significant public health issue due to its prevalence and impact on overall health, well-being, and quality of life, especially in communities where access to dental care and preventive measures is limited.^23,28^ Clinically, it manifests as localized destruction of the tooth surface, resulting in cavities, pain, infection, and tooth loss.^31^ Oral hygiene practices, dietary habits, and the composition and flow of saliva seem to be the most important risk factors for tooth decay.^14,24^ Nevertheless, several less obvious or unknown risk factors can contribute to the development of caries; some of these factors may not be immediately apparent, but they can still increase the risk for tooth decay, gum disease, and tooth loss.^24,28,31^ Also, while advancements in caries diagnosis and treatment are ongoing, limitations in early detection, patient compliance, restoration longevity, and accessibility continue to challenge the effectiveness of caries management. Thus, future improvements should focus on better diagnostic tools, minimally invasive treatments, and cost-effective preventive strategies.^1^

Some studies have shown that dentofacial anomalies, such as malocclusion, can create conditions that increase the risk of tooth decay.^8,11,16^ Malocclusion is a common dental condition characterized by abnormal alignment of the teeth and jaws, with irregularities in tooth position, arch shape, or the occlusal relationship between the maxilla and mandible.^27,38^ Several clinical observational studies have suggested that malocclusion may be associated with increased caries. For example, it has been reported that individuals with malocclusion have a higher likelihood of developing caries.^11,16^ Additionally, a comprehensive study on the connection between orthodontic treatment and the occurrence of new cavities in adolescents observed a tendency for higher caries rates among those receiving orthodontic care.^8^ Mechanistically, it is believed that increased plaque buildup, unbalanced bite, grinding associated with malocclusion, gum disease, difficulty chewing, and the impact of orthodontic appliances are some malocclusion-related factors directly linked with tooth decay.^19^ Besides caries, malocclusion has also been linked to periodontitis.^19^ Some studies have shown that malocclusion and periodontitis are interconnected, as misaligned teeth increase the risk of gum disease, while gum disease can further destabilize teeth. Early diagnosis and proper dental care can help prevent both conditions from causing long-term damage,^4^ further supporting the hypothesis of a correlation between malocclusion and caries. Nevertheless, knowing that various factors, including genetics, can cause malocclusion^25^ and that the observational studies mentioned above have not assessed confounding variables, further investigation on causality is needed.

Mendelian randomization (MR) is a statistical method leveraging genetic variants as IVs (instrumental variables) that offers a powerful tool to infer causality in observational data by mimicking the randomized allocation of exposures seen in clinical trials.^5^ Based on Mendel’s inheritance laws and random gene assortment, MR minimizes confounding factors and reverse causation, offering more reliable causal inference.^29^ The two-sample MR approach, an extension of traditional MR, uses summary data from two separate samples to evaluate the causal link between exposure and outcome, thereby increasing the statistical power and reliability of the analysis.^6^ In this study, a two-sample MR method, leveraging summary statistics from large-scale Genome-Wide Association Studies (GWAS), was used to investigate the potential causal relationship between genetic variations associated with malocclusion and the risk of dental problems (outcomes), including caries, mouth ulcers, toothache, loose teeth, bleeding gums, acute and chronic periodontitis, and painful gums. This study aimed to provide theoretical support for clinical practice and formulating public health policies using the two-sample MR method. Exploring the causal pathways between these two common oral diseases can enhance our understanding of the complex interactions between genetics, oral anatomy, and disease mechanisms, thereby promoting the development of personalized oral healthcare strategies.

## MATERIALS AND METHODS

### Ethics Approval and Consent to Participate

The data for this study were obtained from publicly available databases and published literature and do not require ethical approval and written informed consent.

### Study Design

**Fig 1:**
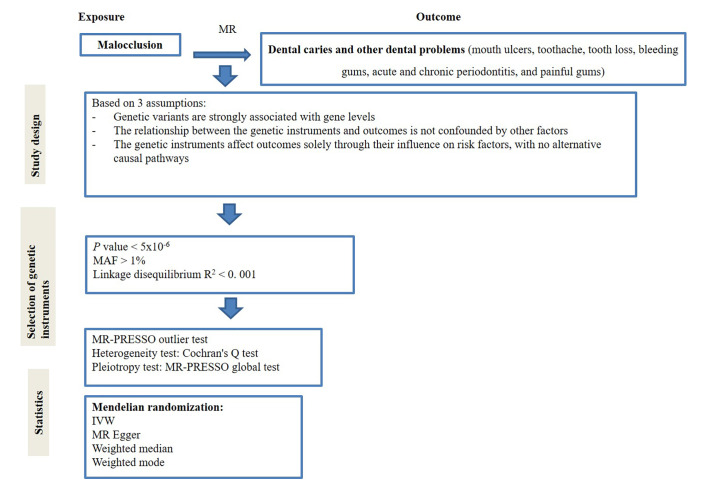
The overall design of the MR analysis framework. MR: Mendelian randomization; IVW: inverse-variance weighted; MR-PRESSO: MR Pleiotropy Residual Sum and Outlier; LOO: leave-one-out; MR-PRESSO; MAF: minor allele frequency.

This study selected genetic IVs in the form of single-nucleotide polymorphisms (SNPs) from GWAS. The MR analysis was conducted according to the three fundamental assumptions:^9,15^ first, genetic variables were statistically significantly associated with the exposure; second, the genetic variants used as instrumental variables for the exposure were not linked to other confounding factors; third, these genetic variants affected the outcome only through their impact on the exposure. Figure 1 provides a detailed overview of the study design.

### Data Sources

The GWAS data for dental problems, including caries, toothache, acute and chronic periodontitis, oral ulcers, painful gums, and tooth loss, were derived from the UKB/GWAS catalog (see Table S1 for more details on the number of cases and controls). Caries status was based on ICD10 K02.9 (dental caries, unspecified) in ukb-e-K02_AFR, on PheCode 521.1 in GCST90044098, and on ICD10-based Phesant-derived variables in ukb-b-4770. For ukb-b-6458 (mouth ulcers), ukb-b-19191 (toothache), ukb-b-12849 (loose teeth), ukb-b-7872 (bleeding gums), and ukb-b-11161 (painful gums). The data were self-reported by the participants through a touchscreen questionnaire, covering oral/dental problems, with the data field being of a multiple-choice categorical type. This provides detailed self-reported records of the prevalence of dental problems in the study population. In GCST90044101, acute periodontitis was based on PheCode 523.31. In GCST90044102, chronic periodontitis was based on PheCode 523.32. The open GWAS exposure data regarding dental anomalies, including malocclusion, were extracted from the FinnGen R12 database (K11_DENTOFACIAL_ANOMALIES; Finland R12: 13,829 cases/310,260 controls) (Table S2). All data were obtained from public registries containing data from studies that already adhered to the Declaration of Helsinki and Good Clinical Practices, and no ethical approval was necessary.

### Selection of Instrumental Variables (IVs)

The process of selecting IVs in this study was as follows (Table S3): (i) we screened the entire genome for SNPs statistically significantly associated with anomalies of malocclusion, initially setting a p-value threshold of 5×10^-8^. Unfortunately, the number of SNPs meeting this stringent criterion was very limited. Thus, to identify more SNPs for study, the inclusion criteria were relaxed to p< 5×10^-6^.^10,35^ (ii) SNPs (minor allele frequency ([MAF]> 0.01) were screened out.^18^ (iii) The linkage disequilibrium (LD) effect among SNPs was eliminated based on the criteria of R^2^ < 0.001 and window size = 10,000kb.^21^ (iv) The F-value was calculated in order to avoid the existence of possible weak IVs. The formula was as follows: F = R^2^×(N-2)/(1-R^2^),^38^ with F > 10.^30^ If the obtained IVs were not present in the summary data of the outcome, proxy SNPs with high LD (R^2^ > 0.8) to IVs were searched through the online platform LD Link for replacement (https://ldlink.nci.nih.gov/). Then, a harmonization process was performed to align the effect alleles of the exposure and outcome SNPs, identifying and excluding SNPs with incompatible alleles and palindromic SNPs with intermediate frequency.

### MR Analysis

The primary analysis utilized the IVW method to calculate the odds ratio (OR) and 95% confidence interval (CI) for assessing the causal relationship between exposure and outcome risk. The IVW method calculates the weighted average effect size by assigning the inverse variance as weights for each SNP.^20,36^ Weighted median,^6^ MR-Egger,^7^ and weighted model^17^ methods were used to validate the results. The MR-Egger method accounts for pleiotropy by considering an intercept term, providing accurate causal effect estimates;^7^ the weighted median method, which assumes that at least half of the instrumental variables are valid, evaluates the causal relationship between exposure and outcome.^6^ All analyses were performed using the “Two Sample MR” package in R version 4.0.5, with visualizations presented in scatter plots and sensitivity analysis plots.

### Sensitivity Analysis

We assessed heterogeneity among instruments using Cochran’s Q test, considering heterogeneity to be low when p > 0.05, indicating that the estimates among instruments were randomly distributed and had little impact on the IVW results.^2^ Additionally, the MR-Egger regression method was used to explore and eliminate the impact of pleiotropy on the estimation of the association due to genetic variation. When the intercept term of the MR-Egger regression approached zero or was not statistically significant, this suggested the absence of pleiotropy. The “leave-one-out” (LOO) analysis was conducted to identify potential heterogeneous SNPs by sequentially omitting each instrumental SNP. Additionally, MR-PRESSO was used to detect and remove potential outliers (SNPs with p < 0.05) and re-estimate the causal associations to correct for potential pleiotropy.^32^ Also, LOO analysis was used to evaluate the presence of predominant Ivs.^34^

## RESULTS

### IVs Screening

When using dentofacial anomalies (including malocclusion) as the exposure, 30 related IVs were selected. The mean of the F-statistic is 22.62, with a minimum value of 20.90 and a maximum value of 27.91 (Table S3). Among those, 64 SNPs did not match information in the summary data, and no proxy SNPs were found, while 7 SNPs had proxy SNPs (Table S3).

### Causal Effect of Dentofacial Anomalies on Dental Caries

The genetic prediction results indicated no statistically significant causal associations between dentofacial anomalies [including malocclusion] and dental caries (Table 1) (all p>0.05). Scatter plots for the relevant causal analyses when assessing cohort 1 (GCST90044098), cohort 2 (ukb-e-K02_AFR), and cohort 3 (ukb-b-4770) are shown in Fig S1, while forest plots for SNP effect analyses are shown in Fig S2.

**Table 1:** MR analysis of the causal relationship between dental anomalies and dental caries

Outcome	nsnp	Method	or_ci	p
Caries (ukb-b-4770)	22	IVW	1.6123 (0.9738 – 2.6694)	0.063
Caries (ukb-b-4770)	22	MR Egger	2.1748 (0.8971 – 5.272)	0.101
Caries (ukb-b-4770)	22	Weighted median	1.9341 (0.9253 – 4.0427)	0.079
Caries (ukb-b-4770)	22	Weighted mode	1.8769 (0.9451 – 3.7273)	0.086
Caries (GCST90044098)	26	IVW	1.0243 (0.8943 – 1.1733)	0.729
Caries (GCST90044098)	26	MR Egger	1.0965 (0.8607 – 1.3969)	0.463
Caries (GCST90044098)	26	Weighted median	1.0517 (0.8566 – 1.2913)	0.63
Caries (GCST90044098)	26	Weighted mode	1.0425 (0.8694 – 1.25)	0.657
Caries (ukb-e-K02_AFR)	14	IVW	0.9997 (0.9989 – 1.0005)	0.472
Caries (ukb-e-K02_AFR)	14	MR Egger	0.9997 (0.9982 – 1.0011)	0.652
Caries (ukb-e-K02_AFR)	14	Weighted median	0.9997 (0.9986 – 1.0008)	0.571
Caries (ukb-e-K02_AFR)	14	Weighted mode	0.9997 (0.9987 – 1.0007)	0.54


The MR-Egger regression results indicated that horizontal pleiotropy did not affect the analysis (Table S4). In addition, Cochran’s Q confirmed no heterogeneity (Table S4), while the MR-PRESSO results suggested no outliers (Table S5). Additionally, the LOO analysis showed that no abnormal SNP statistically significantly influenced the causal estimation results (Fig S3), while the funnel plot confirmed that there was no potential bias in the results (Fig S4). This finding suggests that dentofacial anomalies, including malocclusion, do not have a direct genetic impact on the development of caries.

### Causal Effect of Dentofacial Anomalies on Other Dental Problems

Considering that negative results were obtained when assessing the causal link between dentofacial anomalies and caries, we examined whether there might be a causal association between dentofacial anomalies, including malocclusion, and other health problems. The genetic prediction results indicated no statistically significant causal associations between dentofacial anomalies, including malocclusion, and other health problems, including mouth ulcers, toothache, loose teeth, bleeding gums, acute and chronic periodontitis, and painful gums (Table 2). When assessing the causal link between dentofacial anomalies and loose teeth, analysis revealed heterogeneity (Cochran’s Q p=0.027) and suggested horizontal pleiotropy (MR-Egger intercept p=0.045). These findings indicated the presence of one likely outlier, rs79490532 (Table S4). While MR-PRESSO did not detect outliers for loose teeth (Table S5), rs79490532 were subsequently removed using LOO analysis. Even after removing the outlier, the results for the causal effect remained statistically non-significant (Tables S6-8). The sensitivity analyses showed improved robustness with reduced heterogeneity (Cochran’s Q p=0.03, Table S7) and the MR-Egger intercept becoming non-significant (p=0.067, Table S7), further supporting that rs79490532 was the main driver of the initial warning signals.

**Table 2:** MR analysis of the causal relationship between dental anomalies and other oral problems (before removing outliers)

Exposure	Outcome	nsnp	Method	or_ci	p
Dentofacial anomalies (including malocclusion)	Acute periodontitis	27	IVW	0.839 (0.3853 – 1.8267)	0.658
Dentofacial anomalies (including malocclusion)	Acute periodontitis	27	MR Egger	0.3822 (0.0965 – 1.514)	0.183
Dentofacial anomalies (including malocclusion)	Acute periodontitis	27	Weighted median	1.0712 (0.3906 – 2.9375)	0.894
Dentofacial anomalies (including malocclusion)	Acute periodontitis	27	Weighted mode	0.9581 (0.3608 – 2.5444)	0.932
Dentofacial anomalies (including malocclusion)	Loose teeth	23	IVW	1.0009 (0.9982 – 1.0036)	0.502
Dentofacial anomalies (including malocclusion)	Loose teeth	23	MR Egger	1.005 (1.0005 – 1.0096)	0.041
Dentofacial anomalies (including malocclusion)	Loose teeth	23	Weighted median	1.0024 (0.999 – 1.0057)	0.171
Dentofacial anomalies (including malocclusion)	Loose teeth	23	Weighted mode	1.0026 (0.9995 – 1.0058)	0.112
Dentofacial anomalies (including malocclusion)	Chronic periodontitis	27	IVW	1.1006 (0.871 – 1.3906)	0.422
Dentofacial anomalies (including malocclusion)	Chronic periodontitis	27	MR Egger	0.9879 (0.649 – 1.504)	0.955
Dentofacial anomalies (including malocclusion)	Chronic periodontitis	27	Weighted median	1.0225 (0.7189 – 1.4542)	0.902
Dentofacial anomalies (including malocclusion)	Chronic periodontitis	27	Weighted mode	1.0033 (0.7236 – 1.3911)	0.984
Dentofacial anomalies (including malocclusion)	Toothache	23	IVW	0.9998 (0.9977 – 1.002)	0.89
Dentofacial anomalies (including malocclusion)	Toothache	23	MR Egger	1.001 (0.997 – 1.0049)	0.637
Dentofacial anomalies (including malocclusion)	Toothache	23	Weighted median	1.0002 (0.997 – 1.0033)	0.919
Dentofacial anomalies (including malocclusion)	Toothache	23	Weighted mode	1.0002 (0.9971 – 1.0033)	0.907
Dentofacial anomalies (including malocclusion)	Mouth ulcers	24	IVW	1.0024 (0.9987 – 1.006)	0.2
Dentofacial anomalies (including malocclusion)	Mouth ulcers	24	MR Egger	1.0007 (0.9941 – 1.0074)	0.832
Dentofacial anomalies (including malocclusion)	Mouth ulcers	24	Weighted median	1.0022 (0.9972 – 1.0072)	0.395
Dentofacial anomalies (including malocclusion)	Mouth ulcers	24	Weighted mode	1.0016 (0.9969 – 1.0063)	0.511
Dentofacial anomalies (including malocclusion)	Bleeding gums	25	IVW	0.9986 (0.9948 – 1.0023)	0.454
Dentofacial anomalies (including malocclusion)	Bleeding gums	25	MR Egger	0.9998 (0.9929 – 1.0068)	0.956
Dentofacial anomalies (including malocclusion)	Bleeding gums	25	Weighted median	0.9995 (0.9943 – 1.0047)	0.843
Dentofacial anomalies (including malocclusion)	Bleeding gums	25	Weighted mode	0.9993 (0.9945 – 1.0041)	0.779
Dentofacial anomalies (including malocclusion)	Painful gums	23	IVW	0.9999 (0.9981 – 1.0016)	0.89
Dentofacial anomalies (including malocclusion)	Painful gums	23	MR Egger	1.0007 (0.9976 – 1.0039)	0.652
Dentofacial anomalies (including malocclusion)	Painful gums	23	Weighted median	0.9999 (0.9971 – 1.0028)	0.961
Dentofacial anomalies (including malocclusion)	Painful gums	23	Weighted mode	0.9997 (0.9973 – 1.0022)	0.834


## DISCUSSION

The present MR study investigated the potential causal effects of genetic variations associated with dentofacial anomalies, such as malocclusion, and the risk of dental problems. Our data suggests that dentofacial anomalies, including malocclusion, do not have a direct genetic impact on dental health.

This study first examined the causal link between dentofacial anomalies, such as malocclusion, and the risk of developing caries. So far, several clinical studies have suggested a link between dentofacial anomalies, including malocclusion, and an increased risk of developing dental caries. For example, Gaikwad et al^13^ investigated the prevalence of caries in children with malocclusion and found a positive correlation between caries severity, the Dental Aesthetic Index (DAI), and age. Similarly, Feldens et al^11^ studied 509 adolescents aged 11 to 14 years enrolled in public schools in Osório, southern Brazil, and concluded that handicapping malocclusion, maxillary irregularity, and abnormal molar relationships are associated with both the occurrence and severity of caries. However, both authors agreed that this relationship seems complex and influenced by multiple factors. The aforementioned studies concluded that dental anomalies indirectly contribute to caries development by affecting oral hygiene, function, and microbiology rather than serving as an independent cause. They also emphasized the need for longitudinal and genetic studies to establish a definitive causal link.

Genetic factors play an important role in dentofacial development and susceptibility to caries, and some studies suggest that shared genetic pathways may contribute to both conditions. According to some data, specific genetic loci influence craniofacial development and enamel formation, potentially affecting caries susceptibility.^33^ For example, studies have suggested that variants in AMELX, ENAM, and DSPP genes, which regulate enamel and dentin formation, may contribute to malocclusion and caries risk.^23^ In this study, data on caries were collected from 3 large cohorts. However, we found no link between the two. This finding suggests that dentofacial anomalies, including malocclusion, do not have a direct genetic impact on the development of dental caries. Still, further genomic and longitudinal studies are needed to confirm the extent of this genetic interplay.

Considering that no links were obtained between dentofacial anomalies and caries, we examined whether there might be a causal association between dentofacial anomalies and other health problems. In addition to periodontitis,^19^ existing evidence indicates that dentofacial anomalies can indirectly contribute to the development of oral ulcers, primarily due to mechanical trauma resulting from malocclusion.^12^ Furthermore, studies have also suggested a link between dentofacial anomalies and bleeding gums, mainly due to their impact on oral hygiene, plaque accumulation, and gingival health. Several mechanisms underlie this association.^3^ However, the direct effect of dentofacial anomalies has not yet been explored. Our data suggests that dentofacial anomalies, including malocclusion, do not have a direct genetic impact on the development of dental problems.

The present study has several limitations that should be pointed out. First, the study was constrained by the limited availability of independent SNPs related to some of the exposure factors, which could have limited the power to detect causal associations. Secondly, the study focused solely on assessing the causal influence of dentofacial anomalies (including malocclusion) and dental deposits on the risk of dental problems, neglecting the possibility of reverse causation or bidirectional relationships. Third, the FinnGen K11 definitions are an important point that affects the specificity of the exposure “dentofacial anomalies, including malocclusion”. Using FinnGen K11 (a broad category) as the exposure has limitations, since this classification includes other dentofacial anomalies beyond malocclusion. Unfortunately, data with a finer resolution are not available for the time being, since the data were limited by what was available in the data repositories. Nevertheless, this broad definition may introduce heterogeneity and probably dilute the true effect of a specific subtype (e.g., isolated malocclusion) or cause the IVs to capture genetic signals related to other anomalies. Future GWAS studies should refine the data – specifically on malocclusion – which would help more accurately assess the causal relationship between malocclusion and dental problems. Fourth, the IV selection threshold had to be relaxed to 5×10⁻⁶. Admittedly, relaxing the inclusion threshold is not ideal, but it is necessary because of insufficient SNPs to perform the analysis. The F-values indicated the absence of weak instrument bias. Therefore, despite relaxing the threshold, the IVs still exhibit sufficient strength, mitigating potential biases associated with weak instruments. Finally, we acknowledge the limited generalized applicability of these results, as the population studied was a single country (northern European dataset).

## CONCLUSION

Thess finding suggest that dentofacial anomalies, including malocclusion, do not have a direct genetic impact on dental health. However, future work should delve into the underlying mechanisms and clinical applications, focusing more on modifiable pathogenic pathways, such as effective brushing and diet, rather than occlusal abnormalities.

### REFERENCES

**Fig S1:** Scatter plot images. The causal effect of dentofacial anomalies, including malocclusion, on caries. Data based on (a) Cohort 1 (GCST90044098); (b) Cohort 2 (ukb-e-K02_AFR); (c) Cohort 3 (ukb-b-4770).

**Fig S2:** Forest plot images. The causal effect of dentofacial anomalies, including malocclusion, on caries. Data based on (a) Cohort 1 (GCST90044098); (b) Cohort 2 (ukb-e-K02_AFR); (c) Cohort 3 (ukb-b-4770).

**Fig S3:** LOO plot images. The causal effect of dentofacial anomalies, including malocclusion, on caries. Data based on (a) Cohort 1 (GCST90044098); (b) Cohort 2 (ukb-e-K02_AFR); (c) Cohort 3 (ukb-b-4770).

**Fig S4:** Funnel plot images. The causal effect of dentofacial anomalies, including malocclusion, on caries. Data based on (A) Cohort 1 (GCST90044098); (B) Cohort 2 (ukb-e-K02_AFR); (C) Cohort 3 (ukb-b-4770).

**Table S2:** Data regarding exposure factors

Trait	Exposure	GWAS ID	Sample Size (case/control)
Dentofacial anomalies [including malocclusion]	Dentofacial anomalies [including malocclusion]	K11_DENTOFACIAL_ANOMALIES (Finland R12)	13,829/ 310,260


**Table S1:** Data regarding outcomes

Trait	Disease	GWAS ID	Sample Size (case/control)	Number of SNPs
Diagnoses main ICD10 K02.9 Dental caries, unspecified	Dental caries	ukb-e-K02_AFR	239/6,636	15,377,798
Dental caries (PheCode 521.1)	GCST90044098	2,906/453,442	NA
Dental caries	ukb-b-4770	463,010	9,851,867
Mouth/teeth dental problems: Mouth ulcers	Oral ulcers	ukb-b-6458	461,113	9,851,867
Mouth/teeth dental problems: Toothache	Toothache	ukb-b-19191	461,113	9,851,867
Mouth/teeth dental problems: Loose teeth	Loose teeth	ukb-b-12849	461,113	9,851,867
Mouth/teeth dental problems: Bleeding gums	Bleeding gums	ukb-b-7872	461,113	9,851,867
Acute periodontitis (PheCode 523.31)	Acute periodontitis	GCST90044101	456,348	NA
Chronic periodontitis (PheCode 523.32)	Chronic periodontitis	GCST90044102	456,348	NA
Mouth/teeth dental problems: Painful gums	Painful gums	ukb-b-11161	461,113	9,851,867


**Table S8:** Results of MR-PRESSO test (after removing outliers)

Exposure	Outcome	Raw	Outlier corrected	Global P	Number of outliers	Distortion P
OR (CI%)	P	OR (CI%)	P
Dentofacial anomalies [including malocclusion]	Loose teeth	0.9997 ( 0.9962 - 1.0033 )	0.884	NA	NA	0.027	0	NA


**Table S7:** Heterogeneity and pleiotropy tests of instrumental variables (after removing outliers)

Exposure	Outcome	Heterogeneity	Pleiotropy
Q statistic (IVW)	P value	MR-Egger Intercept	P value
Dentofacial anomalies [including malocclusion]	Loose teeth	34.716	0.03	-6.8e-04	0.067


**Table S6:** MR analysis results after removing outliers

Exposure	Outcome	N.SNPs	Methods	OR (95% CI)	P
Dentofacial anomalies [including malocclusion]	Loose teeth	22	IVW	0.9997 ( 0.9962 - 1.0033 )	0.883
	Loose teeth	22	MR Egger	1.0076 ( 0.999 - 1.0163 )	0.1
	Loose teeth	22	Weighted median	1.0016 ( 0.9975 - 1.0057 )	0.44
	Loose teeth	22	Weighted mode	1.0027 ( 0.9964 - 1.0089 )	0.416









